# Association between High Interferon-Gamma Production in Avian Tuberculin-Stimulated Blood from *Mycobacterium avium* subsp. *paratuberculosis*-Infected Cattle and Candidate Genes Implicated in Necroptosis

**DOI:** 10.3390/microorganisms11071817

**Published:** 2023-07-15

**Authors:** Gerard Badia-Bringué, María Canive, Patricia Vázquez, Joseba M. Garrido, Almudena Fernández, Ramón A. Juste, José Antonio Jiménez, Oscar González-Recio, Marta Alonso-Hearn

**Affiliations:** 1 Department of Animal Health, NEIKER-Basque Institute for Agricultural Research and Development, Basque Research and Technology Alliance (BRTA), 48160 Derio, Spain; 2 Doctoral Program in Molecular Biology and Biomedicine, Universidad del País Vasco/Euskal Herriko Unibertsitatea (UPV/EHU), 48940 Leioa, Spain; 3 Departamento de Mejora Genética Animal, Instituto Nacional de Investigación y Tecnología Agraria y Alimentaria (INIA), Consejo Superior de Investigaciones Científicas, 28040 Madrid, Spain; 4 CONAFE—Spanish Confederation of Holstein Cattle, 28009 Madrid, Spain

**Keywords:** *Mycobacterium avium* subsp. *paratuberculosis*, necroptosis, apoptosis, interferon, disease resistance, innate immune response

## Abstract

The mechanisms underlying host resistance to *Mycobacterium avium* subsp. *paratuberculosis* (MAP) infection are largely unknown. In the current study, we hypothesize that cows with an ability to produce higher levels of interferon-gamma (IFNɣ) might control MAP infection more successfully. To test this hypothesis, IFNɣ production was measured using a specific IFNɣ ELISA kit in avian purified protein derivative (aPPD)-stimulated blood samples collected from 152 Holstein cattle. DNA isolated from peripheral blood samples of the animals included in the study was genotyped with the EuroG Medium-Density Bead Chip, and the genotypes were imputed to whole-genome sequencing. A genome-wide association analysis (GWAS) revealed that high levels of IFNɣ in response to the aPPD were associated with a specific genetic profile (heritability = 0.64) and allowed the identification of 71 SNPs, 40 quantitative trait loci (QTL), and 104 candidate genes. A functional analysis using the 104 candidate genes revealed a significant enrichment of genes involved in the innate immune response and, more specifically, in necroptosis. Taken together, our results define a heritable and distinct immunogenetic profile associated with the production of high IFNɣ levels and with the capacity of the host to lyse MAP-infected macrophages by necroptosis.

## 1. Introduction

Paratuberculosis (PTB) is a chronic granulomatous enteritis that affects domestic and wild ruminants worldwide. It is caused by *Mycobacterium avium* subsp. *paratuberculosis* (MAP), an intracellular, Gram-positive pathogen. The economic impact of PTB on the dairy industry has been estimated between USD 250 million and USD 1.5 billion annually in the US, and at USD 364.31 million per year in Europe [1]. In Europe and North America, PTB is endemic in dairy cattle, with herd prevalence estimates higher than 50% [2]. PTB-associated losses include increased susceptibility to other diseases, increased somatic cell counts, increased incidence of clinical mastitis, reduced fertility, reduced milk production, and involuntary culling of cows [3]. In addition, several studies have detected MAP presence in the intestines of patients with Crohn’s disease (CD), ulcerative colitis (UC), and idiopathic inflammatory bowel disease (IBD)-associated colorectal cancer [4,5,6,7]. Moreover, there is evidence suggesting that MAP infection is associated with several human autoimmune diseases and Alzheimer’s disease [8]. Currently, there is no commercial treatment for bovine PTB, and the parenteral vaccination with heat-killed inactivated vaccines is not widely accepted by animal health authorities on the grounds of a slight interference with the diagnosis of bovine tuberculosis [9]. Current control programs are based on the implementation of biosecurity measures and test and culling strategies. However, the efficacy of this last strategy is hampered by the low sensitivity of current methods for the detection of subclinical MAP infections [10]. One complementary solution to control MAP infection is the identification of host genetic variants determining variation in immune-related traits that could be used to identify and select resistant animals. Although this is a medium- or long-term strategy, its effects are permanent, transferred to the subsequent generations, and can result in disease eradication.

Once ingested, MAP reaches the jejunum and ileum and crosses the intestinal mucosa by binding to fibronectin β1 receptors present on M cells located in Peyer’s patches and is then engulfed by subepithelial macrophages [11]. MAP’s intracellular survival within macrophages is achieved by inhibiting the maturation and acidification of the phagosome and preventing the presentation of antigens to the immune system [12,13,14,15]. Triggered upon MAP infection, some cellular pathways can be activated in an attempt by the host to counteract MAP survival within infected macrophages. Infected macrophages express the major histocompatibility complex II (MHCII) and secrete interleukin 12 (IL12), interleukin 18 (IL18), and interleukin 8 (IL8). Consequently, T and B lymphocytes are recruited and activated at the site of the infection. After activation, Th1 lymphocytes secrete interferon-gamma (IFNγ), which recruits and activates new macrophages, resulting in IL12 and tumor necrosis factor (TNFα) production [16]. Previous studies have proven that the host’s ability to express IFNɣ is crucial for the control of mycobacterial infections [17,18].

PTB is a multifactorial disease that is the result of the interaction of genetic, environmental, and microbial factors. A possible explanation of the differences between cows in the disease outcome could be the presence of certain genetic profiles that could modify the immune response against MAP, thus making some cows more resistant than others. Host genetics play an important role in the susceptibility/resistance to MAP infection; therefore, a breeding strategy focused on increasing resistance to MAP infection and on reducing susceptibility is feasible and currently implemented in some countries [19,20,21,22]. By performing a genome-wide association analysis (GWAS), our research group has recently identified a total of 398 single-nucleotide polymorphisms (SNPs) associated (FDR ≤ 0.05) with a combination of antemortem (serum ELISA) and postmortem (tissue PCR and culture) PTB diagnostic definitions in a common set of Spanish Holstein cattle (N = 983) using whole-genome sequence (WGS) data [19]. We were also able to identify 449 and 752 SNPs associated with the presence of multifocal or diffuse PTB-associated lesions, respectively [23]. Next, we demonstrated that there is genetic variation associated with PTB tolerance using WGS data and the combination of the results of three diagnostic tests: histopathology, tissue PCR, and bacteriological culture of gut tissues [24]. PTB-tolerant cows were defined as infected animals with positive PCR and bacteriological culture results for the detection of MAP but without lesions in gut tissues. In addition, the tolerant cows did not show PTB-associated clinical signs. However, the use of gut tissues from slaughtered animals is required to detect MAP by PCR and bacteriological culture, and to perform the histopathological analysis which limits the use of this phenotype as an indicator (“proxy”) of disease tolerance.

Defining the adequate phenotype is the main challenge in identifying the genetic profile of resistance against MAP infection. Reductionist approaches investigate a host biological subsystem, such as a key cellular function, whose performance is the criterion for the classification of the population [25]. Recently, a heritable and distinct immunogenetic profile in MAP-infected macrophages designed to limit bacterial load early after infection was described [26]. Some of the identified candidate genes, such as oxysterol-binding protein-like 6, cysteine- and serine-rich nuclear protein 3, and coiled-coil domain-containing 92, regulate cellular cholesterol trafficking and efflux, apoptosis, and IFN production, respectively. Similarly, the measure of indicators of T-lymphocyte performance in response to MAP infection, such as IFNɣ production, could be an appropriate strategy for the identification of resistant cattle. In the current study, therefore, we hypothesized that animals able to induce a strong and early innate immune response mediated by IFNɣ might limit MAP load within macrophages better than others and that a considerable proportion of this interindividual variability might be genetic. Our goal was to identify SNPs, quantitative trait loci (QTLs), candidate genes, and mechanisms associated with resistance to MAP infection using IFNɣ production as an indicator. Since there is evidence that allelic variants affecting genes involved in the innate immune responses may contribute to resistance to multiple pathogens [27], the identified QTLs and candidate genes were compared with QTLs and candidate genes for other bovine diseases and health and fertility traits. In addition, the candidate genes identified in our study were compared with human candidate genes previously identified in CD, UC, IBD, colorectal cancer, and tuberculosis. Before the identified SNPs can be used to guide selection, undesirable genetic linkages with other traits and phenotypes included in the Spanish Holstein cattle evaluations were assessed. For this purpose, estimated breeding values (EBVs) for IFNɣ production were estimated in a larger independent population (N = 1739) and correlated with 65 traits and phenotypes included in the Spanish evaluations of Holstein cattle.

## 2. Materials and Methods

### 2.1. Animals and Disease Status

Friesian cattle included in this study were not submitted to any in vivo experimentation; therefore, no specific ethics committee authorization was needed. The cows were slaughtered in the Bilbao and Donostia municipal slaughterhouses (Basque Country, Spain) under the pertinent Basque (Basque Government Decree 454/1994), Spanish (Spanish Government Law 32/2007 and Royal decree 731/2007), and European (Council Regulation No1099/2009) legislation on animal welfare. The animals selected for the study consisted of 152 culled Holstein cattle from several herds located in seven Spanish regions: Basque Country (N = 45), Catalonia (N = 67), Navarre (N = 1), Cantabria (N = 30), Aragon (N = 1), Castile and Leon (N = 5), and Asturias (N = 1), along with two slaughtered cows of unknown origin. All cows were older than 2 years. These animals belonged to a reference population of 986 culled Holstein cattle that were slaughtered from March 2007 to May 2010. In each weekly visit to the abattoirs, 2–6 Holstein cows older than 2 years were sampled. The infection status of the 986 animals was determined by ELISA for the detection of MAP antibodies, PCR for the detection of MAP DNA in gut tissues, and histopathological analysis of gut tissues as previously described [28]. For the histopathological analysis, samples from the distal jejunum, ileocecal valve (ICV), and jejunal and ileal lymph nodes were collected and fixed in 10% neutral buffered formalin, dehydrated through alcohol gradient, and embedded in paraffin wax using standard procedures. Samples were then cut into 4 µm sections using a microtome and stained with hematoxylin–eosin (HE) and Ziehl–Neelsen (ZN). The stained sections were examined by light microscopy to classify samples into four groups: without lesions and with focal, multifocal, or diffuse lesions [29].

### 2.2. Interferon-Gamma Release Assay (IGRA)

IGRA was performed in blood samples from only 343 of the 986 culled cows. Blood stimulation was performed within the first 8 h after blood collection. Briefly, four 1.4 mL aliquots of lithium heparinized whole blood samples from each animal were added to four wells of a 24-well plate (Becton Dickinson, Franklin Lakes, NJ, USA). The blood samples were then stimulated with 100 µL of phosphate-buffered saline (PBS), 100 µL of avian purified protein derivative (aPPD) (0.3 µg/µL) (CZ Veterinaria^®^ SA, Porriño, Spain), 100 µL of bovine purified protein derivative (bPPD) (0.3 µg/µL) (CZ Veterinaria^®^ SA, Porriño, Spain), and 100 µL of lectin (1 μg/mL) as a positive control. Avian tuberculin PPD stimulating antigen contains a purified protein derivative prepared from the filtrate of a heat-killed *Mycobacterium avium* (strain D4ER) grown on a synthetic medium. Bovine tuberculin PPD is derived from *Mycobacterium bovis*, strain AN-5. After incubation for 16–24 h at 37 °C in a 5% CO_2_ incubator, the plasmas were separated by centrifugation at 500× *g* for 10 min at room temperature (RT) and then frozen at −20 °C until testing. Subsequently, IFNɣ levels were measured in triplicate in the plasma samples using a specific IFNɣ ELISA test according to the manufacturer’s instructions (Bovigam^TM^, Prionics, Schlieren, Switzerland). Briefly, 50 µL of plasma diluent and 50 µL of each stimulated sample were pipetted into a 96-well plate, and the plate was incubated at RT for 60 min. The plate was then washed six times, 100 µL of the conjugate antibody was added, and the plate was incubated at RT for 60 min. After six more washes, 100 µL of the chromogen solution was added, and the plate was incubated for 30 min at RT. Finally, 50 µL of STOP solution was added, and the plate was read at 650 and 450 nm (final OD value = OD_450_ − OD_650_). IFNɣ levels were expressed as the OD of the aPPD or bPPD-stimulated plasmas minus the OD of the PBS-stimulated samples. The 152 cows from the 343 animals with an IGRA record were selected because they had an OD (aPPD − PBS) higher than the OD (bPPD − PBS), and the OD (aPPD) was also higher than the OD (PBS). This selection avoids false-positive reactions with *Mycobacterium bovis* infection and negative OD values, respectively. Three of the 152 animals with IGRA results did not have histopathology results. According to the interpretation criteria of the kit, 106 of the 152 cows included in the study were PTB-positive because the OD of the aPPD − PBS was higher than 0.05, and the OD (aPPD − PBS) was higher than the OD (bPPD − PBS). None of the 152 cows were tuberculosis-positive according to the IGRA kit interpretation criteria.

### 2.3. Genotyping and Imputation to Whole-Genome Sequence (WGS)

Peripheral blood (PB) samples were collected into 10 mL Vacutainer EDTA tubes (Becton Dickinson, Franklin Lakes, NJ, USA) from the 152 cows of the study population at the time of slaughter. Total DNA was extracted from the PB samples using the QIAmp DNA Blood Mini Kit according to the manufacturer’s instructions (Qiagen, Hilden, Germany). Purified DNA was then quantified by spectrophotometry and genotyped using the EuroG Medium-Density Bead Chip (Ilumina, San Diego, CA, USA) at the molecular genetic laboratory service of the Spanish Federation of Holstein Cattle (CONAFE) using the *InfiniumTM iScan* system for allele assignation (Illumina, San Diego, CA, USA). The individual genotypes were imputed to WGS as previously described [19]. Briefly, genotypes were phased using *Eagle2.-4.* [30] and imputed with *minimac4 1.0.2* [31] to the Bovine High-Density Bead Chip (581,712 SNPs) using a reference panel of 1278 Holstein bulls from Run7.0 of the 1000 Bull Genomes project and 581,712 SNPs (ARS-UCD1.2) Imputation to the WGS level (ARS-UCD1.2) was then undertaken using the same phasing and imputation procedure and a reference population of 2333 *Bos taurus* from Run7.0 of the 1000 Bull Genomes project [32]. Finally, the following filters were applied: call rate > 0.80, minimum allele frequency (MAF) > 0.01, and imputation score (r^2^) > 0.7. The final number of SNPs per animal was 12,856,161.

### 2.4. GWAS Analysis, Variance Components, and h^2^ Estimation

The IFNɣ levels in response to the aPPD, OD (aPPD − PBS), represented the quantitative phenotype in the GWAS analysis. The variance components and heritability (*h*^2^) explained by all the SNPs were calculated using the genome-wide complex trait analysis (*GCTA1.93.2* [33], according to the following formula:

$$h^{2}=\frac{\sigma_{G}^{2}}{\sigma_{G}^{2}+\sigma_{e}^{2}},$$

where $\sigma_{G}^{2}$ represents the variance explained by all the SNPs, and $\sigma_{e}^{2}$ is the residual variance. The WGS data and the OD of the aPPD − PBS data were analyzed using the mixed linear model association analysis of the *GCTA1.93.2* software, expressed as *y* = *a* + *bx* + *g* + *e*. In the model, *y* is the phenotype, *a* is the mean term, *b* is the allele effect, *x* is the genotype of the SNP coded as 0, 1, or 2 depending on how many copies of the minor allele have the animal, *g* is the polygenic effect as a random effect (assumed to be distributed as N ≈ (0,${\;\sigma }_{e}^{2}$), and *e* is the residual effect (also assumed to be distributed as N ≈ (0,${\;\sigma }_{e}^{2}$)). Age was included as a covariate in the analysis. To account for multiple testing, a 5% genome-wide false discovery rate (FDR) was used. A threshold of *p*-value ≤ 5 × 10^−7^ was used as suggested by the Wellcome Trust Case Control Consortium [34]. The inflation factor (*λ*) and quantile–quantile plots were used to compare the observed distributions of the −log (*p*-values) to the expected distribution under the no-association model. A *λ* value close to 1 suggests appropriate adjustment for potential substructure, and *λ* > 1.2 suggests population stratification. The SNP effects (*b*-values) were also calculated using the *GCTA1.93.2* software. If the sign of the *b*-value is positive, it implies that there is a positive relationship between the variables, i.e., SNP and IFNɣ levels.

### 2.5. GWAS Data Postprocessing

Results from the GWAS analysis were filtered by clumping. Briefly, clumping is a process that first selects the most significant SNP and clumps (removes) other SNPs in a particular window that are in linkage disequilibrium with the selected SNP. The clumping was performed using the software *PLINK1.9* [35] with a window of 500 kbp and a linkage disequilibrium-based correlation index (r^2^) of 0.9.

### 2.6. SNPs, Quantitative Trait Loci (QTLs), and Candidate Genes Identification

After the clumping, the localization of the significant SNPs (FDR ≤ 0.05, *p*-value ≤ 5 × 10^−7^), QTLs, and candidate genes was performed using the ARS-UCD1.2 reference genome as previously described [24]. The genomic localization of the identified SNPs in the ARS-UCD1.2 reference genome was determined using the Ensembl Variant Effect Predictor (VEP) [36]. QTLs were defined on the basis of significant SNPs within 500 kbp of each other.. Overlapping QTLs were merged and considered as a single QTL. Candidate genes were identified 50 kbp upstream and downstream of the most upstream and downstream SNPs in the QTL using Ensembl [36]. The identified QTLs and candidate genes were compared with reported and annotated QTLs and candidate genes associated with health, longevity, immunity, milk content, and fertility traits [37]. For a direct connection between the identified QTLs and annotated QTLs for other traits that overlap those regions, a QTL enrichment analysis was performed using the GALLO package [38]. This QTL enrichment analysis is based on a hypergeometric approach where the number of identified QTLs is compared with the number of annotated QTLs for each trait in the reference database. In addition, the identified candidate genes were also compared with human candidate genes previously identified for CD, UC, IBD, colorectal cancer, and human tuberculosis [39]. The function of the candidate genes was searched in GeneCards [40] by searching their gene symbol. To further investigate the function of the identified candidate genes, we searched the Innate DB database [41].

### 2.7. Gene Ontology (GO) and Pathway Enrichment Analysis

Candidate genes were investigated for significant enrichment of Kyoto Encyclopedia of Genes and Genomes (KEGG) pathways using the ClusterProfiler Bioconductor library in R [42,43]. The candidate genes with roles in the innate immune response were analyzed for significant enrichment of pathways with *STRING11.5*. To account for multiple testing, only terms with FDR less than 0.05 were considered significant.

### 2.8. Estimated Breeding Values (EBVs) for IFNγ Production

EBVs for IFNγ production and the effect of the of the 310 SNPs with evidence of association with the IFNγ production (FDR ≤ 0.05) were calculated for each animal in the study population using the genomic best linear unbiased prediction (gBLUP) model of *GCTA 1.93.2*. [44]. Subsequently, EBVs for IFNγ production were predicted in a larger population of 1739 Friesian cattle on the basis of the effect of the 310 SNPs.

### 2.9. Statistical Analysis

An unpaired Student *t*-test with the Welch–Satterthwaite correction was used to compare the IFNγ levels between animals with distinct PTB-associated lesions versus control cows without lesions (GraphPad Prism 8, SanDiego, CA, USA). Associations between the number of animals with the highest IFNγ levels and positive ELISA and PCR results in gut tissues were analyzed with a Fisher exact test using *jamovi2.3* [45]. Differences were considered significant when *p*-values were less than 0.05. Correlations between the EBVs for IFNγ levels and 65 phenotypes and traits included in the evaluations of Spanish Holstein cattle were calculated in a larger population (N = 1739) with the Spearman’s rank correlation (ρ) implemented in *R4.1.2*.

## 3. Results

### 3.1. Assessment of IFNɣ Production

**Figure 1:**
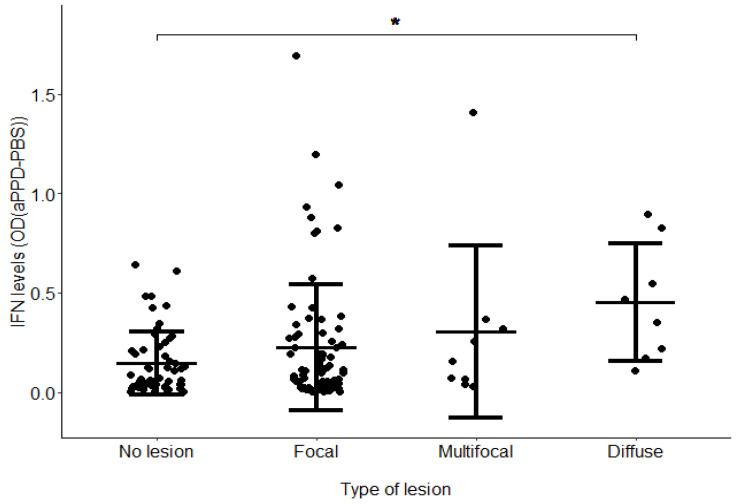
IFNγ production. Error plots of the levels of IFNɣ in aPPD-stimulated blood samples from cows without lesions (N = 56), or with focal (N = 76), multifocal (N = 9), or diffuse (N = 8) lesions in gut tissues. The central line represents the mean of each group, and the whiskers represent the standard error. Significant differences between the cows without lesions (mean = 0.14) and with diffuse lesions (mean = 0.45) were observed (*p* = 0.023). * *p* ≤ 0.05.

IFNɣ levels after blood stimulation with aPPD (OD (aPPD − PBS)) took values between 0.001 and 1.693, with most of the animals showing OD values between 0.001 and 0.750 (Supplementary Table S1). Figure 1 shows the mean of the IFNɣ production after blood stimulation with aPPD (OD (aPPD − PBS)) according to the lesional category.

The mean OD (aPPD − PBS) of the population was 0.217, and the highest value was 1.693. According to the interpretation criteria of the kit, 106 of the 152 cows included in the study had an IGRA-positive result. From the 56 animals without lesions, 35 showed a positive IGRA result. Those animals could be infected with MAP but did not have visible histopathological lesions at the time of slaughter. Significant differences between the cows without lesions (mean = 0.14) and with diffuse lesions (mean = 0.45) were observed (*p*-value = 0.023). No statistically significant differences between the means of the other groups were found. The highest IFNγ levels corresponded to 12 cows with IFNγ levels that were two standard deviations above the mean and with diffuse (2/12), multifocal (1/12), and focal (8/12) lesions.. Interestingly, 50% (6/12) of the animals with the highest IFNγ levels had a positive PCR result for the detection of MAP DNA in gut tissues while only 16% (23/140) of the remaining animals had a PCR positive result (*p*-value = 0.023). This result suggests that the highest IFNγ levels are significantly associated with the presence of MAP in gut tissues. No statistical differences were observed between high IFNγ levels and ELISA-positive results.

### 3.2. Heritability (h^2^) Estimate, Variance Components, and GWAS Results

**Figure 2:**
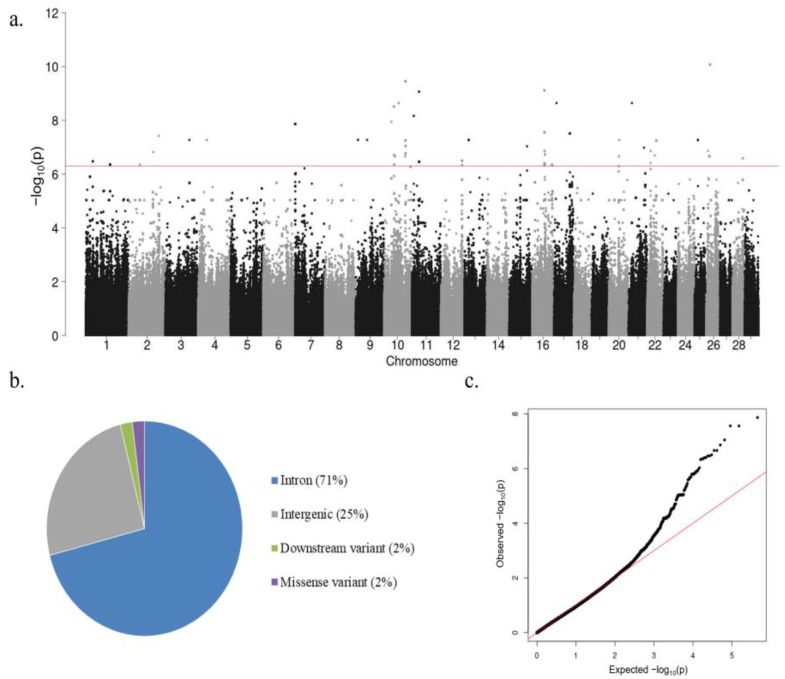
Results of the GWAS analysis. (**a**) Manhattan plot of the −log_10_ of the *p*-values of the association test between each SNP and the IFNɣ levels. Each dot represents one SNP. Chromosome localization of the SNPs is indicated on the *x*-axis. The horizontal red line is drawn at −log_10_ (5 × 10^−7^). (**b**) Genomic distribution of the 71 SNPs surpassing the threshold (*p*-value ≤ 5 × 10^−7^) according to the Ensembl Variant Effect Predictor (VEP). (**c**) Quantile–quantile plot comparing the observed distribution of −log (*p*-values) to the expected values under the null hypothesis.

The associations between genome-wide imputed SNPs and IFNɣ levels after blood stimulation with aPPD (OD (aPPD – PBS)) (N = 152) were analyzed using *GCTA1.93.2*. The heritability and variance components of the IFNɣ levels in the study population were estimated as 0.645 (σ_G_ = 0.0546, σ_e_ = 0.0301). The Manhattan plot showing −log_10_ (*p*-values) of the association test between IFNɣ levels and each SNP is represented in Figure 2a.

The total number of SNPs that surpassed the significance criteria (FDR ≤ 0.05) was 310. After clumping, 71 SNPs (*p*-value ≤ 5 × 10^−7^) located on 19 different Bos taurus chromosomes remained. This suggests that IFNɣ production in response to aPPD is a polygenic trait depending on many SNPs located in different chromosomes that altogether can explain approximately 64% of the variance of the phenotype. As seen in Figure 2b, most of the 71 SNPs were in intronic regions (71%), while the remaining identified SNPs were in intergenic regions (25%) or were downstream (2%) or missense (2%) variants. A quantile–quantile plot comparing the observed distribution of −log (*p*-values) to the expected *p*-values under the null hypothesis is shown in Figure 2c. The median inflation factor was 1.001886, which indicates the absence of population stratification.

### 3.3. SNPs, QTLs, and Candidate Genes Associated with High IFNγ Production

After clumping, a total of 71 SNPs, 40 QTLs, 103 candidate genes, and one microRNA were associated with the production of high IFNγ levels after stimulation with aPPD. *p*-values, QTLs positions, and candidate genes within each QTL are presented in Table 1.

The identified QTLs were distributed along the *Bos taurus* genome, with chromosome 10 harboring the highest number of SNPs (N = 17). The QTL that harbored the most significantly associated SNP was in chromosome 26 (*p*-value = 8.37 × 10^−11^), while the SNPs with the stronger effects were in chromosomes 10, 15, 17, and 21 (b-values = 1.338). The b-values of all the identified SNPs were positive, which suggests that all variants were associated with high IFNɣ levels.

**Figure 3:**
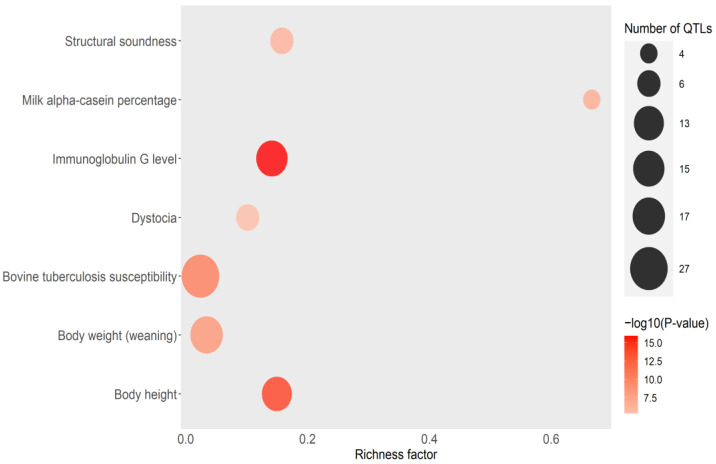
Bubble plot displaying the QTL enrichment results for the top seven enriched traits. A darker red shade in the circles denotes more significant enrichment. The area of the circles is proportional to the number of QTLs. The *x*-axis shows a richness factor obtained by the ratio of the number of QTLs annotated and the total number of each QTL in the reference database.

By looking in the cattle genome database, we found that some of the identified QTLs overlapped with QTLs associated with health (tuberculosis susceptibility, infectious bovine keratoconjunctivitis susceptibility, spongiform encephalopathy, bovine respiratory disease susceptibility, clinical mastitis, somatic cell score, gastrointestinal burden, nematode burden, and tick resistance), immunity (IgG levels), length of production life, milk content (milk linoleic acid content), fertility traits (calving ease, fertility index, retained placenta, conceptive rate, and dystocia), and heat tolerance (Supplementary Table S2). The recurrent association of some of the identified QTLs with multiple health, production, and reproductive traits might suggest the presence of complex regulatory genetic mechanisms such as pleiotropy and epistasis among others. Interestingly, an identified QTL in BTA1 overlapped with a QTL associated with PTB susceptibility (QTL:169891) [46], and two QTLs in chromosomes 16 and 25 overlapped with two QTLs previously associated with PTB tolerance [22], among others. A total of 44 traits were found to be enriched in the QTL enrichment analysis with IgG levels, bovine tuberculosis susceptibility, percentage decrease in packed red blood cell volume (PCV) to day 100 and 150 after challenge with trypanosoma, PCV variance, somatic cell score, gastrointestinal nematode burden, bovine spongiform encephalopathy, final PCV, clinical mastitis, and Marfan syndrome-like disease as the enriched health traits (Supplementary Table S3). Additionally, the identified QTLs were also enriched in several production and reproductive traits. The top seven traits identified in the enrichment QTL analysis including tuberculosis susceptibility, IgG levels, and dystocia are presented in Figure 3.

Some of the identified candidate genes were previously associated with several bovine traits, highlighting their importance not only in health but also in fertility and length of productive life. For instance, the protein kinase cGMP-dependent type I (PRKG1) was associated with 39 bovine traits including PTB susceptibility (QTL: 169927) [46], bovine respiratory disease susceptibility, fertility traits (daughter pregnancy rate, first service conception, inseminations per conception), milk content, and several udder traits. The Fms-related receptor tyrosine kinase 4 (FLT4) was associated with 18 bovine traits including length of productive life, somatic cell score, fertility (calving ease, stillbirth), and udder traits. The identified phospholipase A2 group IVF (PLA2G4F) candidate gene was associated with 15 bovine traits including length of productive life, somatic cell score, fertility traits (calving ease, daughter pregnancy rate, and stillbirth), and udder traits. The calpain 3 (CAPN3) gene was associated with 24 QTLs/traits including bovine respiratory disease susceptibility, length of productive life, somatic cell score, fertility traits (calving ease, daughter pregnancy rate, and stillbirth), and udder traits. CCR4-NOT transcription complex subunit 6-like (CNOT6L), E3 ubiquitin-protein ligase NEDD4, and Acyl-CoA thioesterase 7 (ACOT7) were all associated with the somatic cell score. The identified inter-alpha-trypsin inhibitor heavy chain 5 (ITIH5) gene was associated with bovine respiratory disease susceptibility.

In addition, the identified candidate genes were also compared with human candidate genes previously identified for CD, UC, IBD, colorectal cancer, and tuberculosis. Interestingly, some candidate genes identified in our study, interleukin 18 receptor 1 (IL18R1) and interleukin 1 receptor like-1 (IL1RL1), were also found associated with CD, UC, and IBD. The interleukin 7 receptor (IL7R) was identified as a candidate gene associated with high IFNɣ production, as well as with UC and IBD. NEDD4, CNOT6L, and DNA repair protein RAD51 homolog 2 (RAD51B) were previously associated with IBD, tuberculosis, and colorectal cancer, respectively. From the 83 candidate genes with a recognized gene symbol, 57 (69%) were included in the InnateDB database and had a role in signaling pathways involved in the innate immune response of bovines to microbial infections including IL18R1, IL1RL1, IL7R, Ankyrin repeat domain-containing protein 1 acetylserotonin O-methyltransferase-like (ANKRD1), CD44 antigen precursor (CD44), two integrins (insulin-like growth factor 2 mRNA-binding protein 3 and integrin beta-like protein 1 precursor), an activator of the nuclear factor kappa B (NFKB1) signaling pathway (Pleckstrin homology and RhoGEF domain-containing G5, PLEKHG5), the mitogen-activated protein kinase 9 (MAPK9), the TNF receptor superfamily member 25 (TNFRSF25), and the TNF receptor-associated factor 5 (TRAF5). When the 57 candidate genes with roles in the innate immune response were analyzed with the *STRING11.5*, a significant enrichment (FDR ≤ 0.05) of the necroptosis pathway (bta04217) was observed with five candidate genes belonging to this route (TRAF5, MAPK9, SLC25A6, PLA2G4F, and PLA2G4D).

### 3.4. Gene Ontologies (GOs) and KEGG Pathway Enrichment Analysis

To identify common biological functions in the 104 candidate genes, an enrichment analysis of metabolic pathways was performed using ClusterProfiler. Our results revealed a total of 15 enriched pathways (adjusted *p* ≤ 0.05). As seen in Table 2, necroptosis (bta04217), platelet activation (bta04611), and Ras signaling (bta04014) were found enriched among others. All these pathways had in common the cytosolic phospholipase A2 group IV family (PLA2G4E, PLA2G4D, and PLA2G4F).

### 3.5. EBVs for IFNγ Production and Correlations with Other Bovine Traits

EBVs for IFNγ production were predicted in a larger population of 1739 Friesian cattle. Correlations between the EBVs for IFNγ levels in this population and 65 phenotypes and traits included in the evaluations of Spanish Holstein cattle were calculated with Spearman’s correlation test. Statistically significant and positive correlations were estimated between the EBVs and the protein content in milk (ρ = 0.057), foot health index (ρ = 0.084), combined genetic index (ICO) (ρ = 0.082), longevity (ρ = 0.075), open days (ρ = 0.065), and all the economical indices included in the Spanish evaluations of Friesian cattle. All of these correlations were lower than ρ = 0.2.

## 4. Discussion

Understanding the host–pathogen interaction is key to advancing the development of effective control strategies against MAP infection. There is still a significant gap in our understanding of the protective mechanisms triggered after MAP exposure which might be variable depending on the specific host genetics. Even though the cellular immune response is crucial in the control of intracellular infections, the use of cellular immunity traits in genetic linkage studies in Holstein cattle is scarce. Recently, a strong effect of host genetics on the control of MAP load within infected monocyte-derived macrophages (MDMs) infected with MAP ex vivo was described [26]. Similarly, the measure of indicators of T-lymphocyte performance in response to MAP infection, such as IFNγ production, could be an appropriate strategy for the identification of resistant cattle. There is only one single member representing the Type II IFN group, which is IFNγ. IFNɣ is secreted by T cells, i.e., not directly by MAP-infected cells. IFNɣ binds to its respective receptors (IFNGR1 and IFNGR2) and signals through Janus kinases (JAK1 and JAK2), promoting the phosphorylation and homodimerization of signal transducer and activator of transcription 1 (STAT1), and leading to the expression of IFNɣ activation site (GAS)-regulated genes such as IL18, IL12, and IL27 [47]. IFNɣ is also the main cytokine that drives MHCII’s expression, which highlights the anti-inflammatory action of IFNɣ. In addition to playing a role in both innate and adaptive immune responses against pathogens and tumors, IFNɣ is also important in maintaining immune homeostasis. Consequently, IFNɣ immunotherapy is often used to treat human chronic granulomatous disease, cancer, tuberculosis, cystic fibrosis, hepatitis, osteoporosis, scleroderma, and invasive fungal infections [48].

In the current study, we measured the IFNγ production in response to the aPPD in 152 Holstein cows in two steps consisting of (i) incubating whole blood samples from the selected animals with aPPD, and (ii) detecting the presence of IFNγ released by sensitized lymphocytes in the whole blood sample to indicate a cell-mediated immune response to the specific antigen. The number of samples used in the current study could be considered small in comparison with traditional GWAS analysis using a larger population. However, the assessment of immunocompetence by measuring IFNɣ levels in stimulated blood is a functional and more controlled trait. Similar reductionist phenotypes such as the assessment of macrophage performance by measuring MAP load within MDMs used only 60 samples [26]. In our study, significant differences were observed in the IFNɣ levels measured in aPPDstimulated blood from cows with diffuse lesions (mean = 0.45) and cows without lesions (mean = 0.14) (*p*-value = 0.023), which suggests that the production of IFNɣ is not sufficient to protect against the most severe PTB forms. Even so, the highest IFNγ levels were significantly associated with the presence of focal lesions and MAP bacilli in gut tissues, which suggests a significant role of IFNγ in cows with focal lesions and with a specific genetic profile.

We showed that high IFNγ production is dependent on host genetics (*h*^2^ = 0.64) and identified 71 SNPs, 40 QTLs, 103 candidate genes, and one microRNA associated with high IFNɣ levels. Some of the QTLs identified in the current study overlapped with QTLs associated with health (tuberculosis susceptibility, infectious bovine keratoconjunctivitis susceptibility, spongiform encephalopathy, bovine respiratory disease susceptibility, clinical mastitis, somatic cell score, gastrointestinal burden, nematode burden, and tick resistance), immunity (IgG levels), length of production life, milk content (milk linoleic acid content), fertility traits (calving ease, fertility index, retained placenta, conceptive rate, and dystocia), and heat tolerance. A QTL identified in our study on BTA1 overlapped with a QTL previously associated with PTB susceptibility (QTL:169891) [46], and two identified QTLs were found previously associated with PTB tolerance [24]: 16:71510469–72510469 and 25:11894597–12930197. The locus in BTA16 contains TRAF5, a member of the TNF receptor-associated factor (TRAF) family. This protein is one of the components of a multiple protein complex that binds TNFα receptor (TNFR) cytoplasmic domains and mediates TNFα-induced activation. The binding of TNFα to TNFR1 triggers multiple signaling pathways, including NF-κB, apoptosis, and necroptosis. TNFα has a tolerizing effect on monocytes, desensitizing these cells to additional TLR stimulation as a regulatory mechanism that limits excessive inflammation [49]. In our study, the analysis of KEGG pathway enrichment showed that there was an overrepresentation within the identified QTLs of candidate genes associated with immune-related pathways (necroptosis and platelet activation), cancer-related pathways (Ras signaling and choline metabolism in cancer), and α-linoleic acid metabolism among others. The common genes found in all these pathways included PLA2G4E, PLA2G4D, and PLA2G4F, three cytosolic phospholipases A2 of the group IV family. It has been previously described that the activity of PLA2G is increased in the presence of Ca^2+^, and that PLA2G preferentially hydrolyzes phospholipids, resulting in the formation of arachidonic acid and lysophospholipids, the precursors of prostaglandins, leukotrienes, and platelet-activated factors [50]. Wang et al. demonstrated that IFNγ induces PLA2G-dependent mitochondria-derived reactive oxygen species (ROS) production, and that the increases in PLA2G activities mediate the anticancer effect of IFNγ against colorectal cancer cells [51]. Moreover, Cekay et al. showed that IFNγ induces necroptosis in apoptosis-resistant cancer cells where caspase activation is suppressed [52], highlighting IFNγ’s antiproliferative/antitumor and immunomodulatory effect.

**Figure 4:**
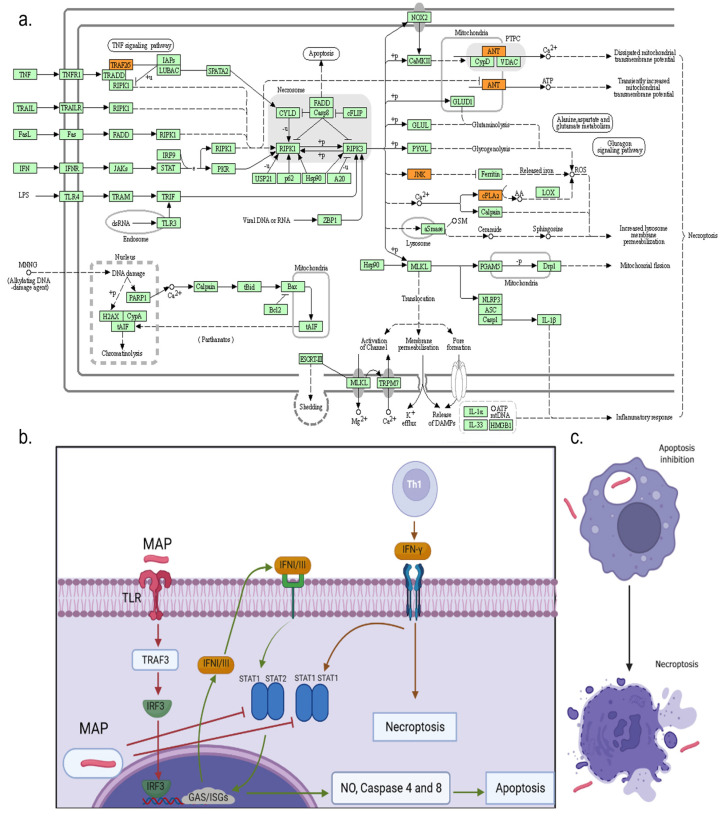
Activation of apoptosis and necroptosis mediated by IFN in MAP-infected macrophages. (**a**) Necroptosis pathway (https://www.genome.jp/pathway/bta04217, accessed on 29 June 2023). Necroptosis can be initiated by different stimuli, such as tumor necrosis factor (TNF), TNF-related apoptosis-inducing ligand (TRAIL), Fas ligand (FasL), interferon (IFN), LPS, viral DNA or RNA, and DNA damage agent, requiring the kinase activity of receptor-interacting protein 1 (RIPK1) and RIPK3. Its execution involves ROS generation, calcium overload, the opening of the mitochondrial permeability transition pore, mitochondrial fission, inflammatory response, and chromatinolysis. Candidate genes identified in our study that are involved in the necroptosis pathway are highlighted in orange: cPLA2—phospholipase 2, ANT—solute carrier family 25 (mitochondrial adenine nucleotide translocator) member 4/5/6/31, JNK—mitogen-activated protein kinase 9 (c-Jun N-terminal kinase), and TRAF5—TNF receptor-associated factor 5. (**b**) Recognition of MAP by Toll-like receptors (TLR) leads to polyubiquitination of TRAF3 (TNF-receptor associated factor 3), which activates IFN regulatory factor 3 (IRF3). The activated IRF3 enters the nucleus and promotes IFNI/III expression and release. Secreted IFNI/III then interacts with their specific receptors. Signal transducer and activator of transcription (STAT1/STAT2) heterodimers binds to enhancer elements which results in the production of proinflammatory and apoptotic factors such as nitric oxide (NO) and caspases 4 and 8. Since MAP blocks apoptosis, an increase in IFNɣ production might trigger necroptosis. (**c**) Cells dying by necroptosis show a necrotic phenotype, including swelling, membrane rupture, and MAP release. Created with BioRender.com.

Emergent evidence strongly supports a role for necroptosis, a recently discovered proinflammatory cell programmed death pathway, in host defense against viral and bacterial pathogens including mycobacteria. Once produced by activated Th1 lymphocytes, IFNɣ is usually secreted to the extracellular environment. In macrophages, IFNɣ binds to its receptor complex composed of IFNGR1 and IFNGR2, and promotes the phosphorylation of STAT1 via JAK1 and JAK2. Phosphorylated STAT1 forms homodimers that bind gamma-activated sequences (GASs) in the nucleus resulting in the induction of proinflammatory cytokines and apoptotic factors such as TNF, IFN types I and III, and caspases 4 and 8 [53,54,55]. IFNs are also known to be potent ROS inducers. In MAP-infected macrophages, however, such signaling pathways are blocked, which effectively inhibits apoptosis. In this context, high IFNɣ levels might result in necroptosis activation. Mediators of IFNɣ-induced necroptosis are Ca^2+^, PLA2, and ultimately ROS [56]. To our knowledge, this is the first study revealing a genetic association between high bovine IFNγ production and host genes involved in necroptosis (Figure 4a). In an environment where apoptosis is inhibited, such as in MAP-infected macrophages, necroptosis could be the most feasible way that certain hosts use to limit MAP intracellular replication (Figure 4b). Similarly, in viral coinfections, necroptosis is a defense mechanism that the host activates in the presence of apoptosis inhibitors [57]. Cells dying by necroptosis show a necrotic phenotype, including swelling and membrane rupture, and release of damage-associated molecular patterns (DAMPs) (Figure 4c).

It was presumed that IFNI and III-driven necroptosis activation might exacerbate inflammation in specific circumstances. In contrast, some studies have demonstrated that necroptosis suppresses inflammation through abruptly terminating cytokine production and cell viability, and that the liberation of endogenous DAMPs is insufficient to cause aberrant inflammation [58]. In human tuberculosis, for instance, necroptosis is primed in *Mycobacterium tuberculosis*-infected macrophages, but macrophage necroptosis is ultimately abrogated, thereby mitigating any pathophysiological role of necroptosis in tuberculosis [59]. Similarly, our results suggest that the differences in the ability of the hosts to induce IFNγ-induced necroptosis after MAP infection may contribute to diverse cattle host responses to MAP infection and disease outcomes. In this context, necroptosis might be beneficial to these hosts able to produce higher levels of IFN and to induce necroptosis by limiting the lifespan of the infected cells. Experimental studies in zebrafish have shown that an excess of TNF also activates necroptosis through mitochondrial ROS production [60].

## 5. Conclusions

We report a new phenotype, the production of high levels of IFNɣ, for evaluating resistance to MAP infection in dairy cows. The SNPs and candidate genes identified revealed an association between high IFNɣ levels after stimulation of blood samples with aPPD and genetics, which helps fill the knowledge gap linking host genetics, innate immune responses, and resistance to MAP infection in cattle. Our results define a heritable and distinct immunogenetic profile associated with the production of high IFNɣ levels and with the ability of the host to lyse infected macrophages by necroptosis. A strong effect of host genetics in producing high IFNɣ levels was observed (*h*^2^ = 0.64) which opens the possibility of ranking Holstein cows on the basis of predicted IFNɣ production. The identified SNPs can be used to develop genetic evaluations for immunocompetence in the Spanish breeding program which would allow producers to select cattle more resistant to MAP infection, and likely for other intracellular pathogens, ultimately reducing the prevalence of diseases and the dependence on antimicrobials, preventing economic losses, increasing the length of cattle productive life, and improving food safety. In addition, our results have important implications regarding the use of the IGRA for the early diagnosis and control of MAP infection. Culling animals producing high levels of IFNɣ could result in the elimination of animals that are controlling MAP load, and that may never shed MAP or show clinical signs of PTB.

## Figures and Tables

**Table 1:** Identified QTLs and candidate genes associated with high levels of IFNɣ.

BTA ^1^	QTL Start (bp)	QTL End (bp)	Peak *p*-Value of Peak SNP	Regression Coefficient (b)	Genes in QTL ^2^	No. of SNPs in QTL
1	26,283,242	26,283,242	3.34 × 10^−7^	0.526	ROBO1	1
1	90,034,825	90,360,896	4.42 × 10^−7^	1.127	5S_rRNA, TBL1XR1, ENSBTAG00000054926	3
2	41,453,339	41,453,339	4.42 × 10^−7^	1.127		1
2	90,850,109	90,850,109	1.51 × 10^−7^	0.474	TBL1XR1, SNORD11B, SNORD11, BMPR2	1
2	111,599,011	111,599,011	3.76 × 10^−8^	0.843		1
3	88,432,545	88,432,545	5.32 × 10^−8^	1.223		1
4	31,901,616	31,901,616	5.32 × 10^−8^	1.223	GPNMB, MALSU1, IGF2BP3, ENSBTAG00000054861	1
7	512,191	941,753	1.36 × 10^−8^	0.720	5S_rRNA, FLT4, CNOT6, GFPT2, MAPK9	5
9	9,107,205	9,107,205	5.32 × 10^−8^	1.223		1
9	42,469,434	42,469,434	5.32 × 10^−8^	1.223	SOBP	1
10	27,643,294	27,643,294	1.13 × 10^−8^	0.854	OR4G10, OR4F67B, OR4G18, OR4K36	1
10	37,469,311	37,833,767	3.07 × 10^−9^	0.811	PLA2G4E, PLA2G4D, PLA2G4F, VPS39, TMEM87A, GANC, CAPN3, ZNF106, SNAP23, LRRC57, HAUS2	3
10	41,108,685	41,108,685	2.18 × 10^−7^	1.105		1
10	54,450,209	54,461,425	2.27 × 10^−9^	1.338	NEDD4, ENSBTAG00000031396	2
10	78,308,107	78,308,107	2.18 × 10^−7^	1.105		1
10	79,501,139	79,957,849	3.55 × 10^−10^	0.839	PLEKHH1, PIGH, ARG2, U6, VTI1B, RDH11, RDH12, ZFYVE26, RAD51B, ENSBTAG00000054736	9
11	7,148,731	7,148,731	6.81 × 10^−9^	1.036	IL1RL1, IL18R1	1
11	26,763,300	26,794,996	8.58 × 10^−10^	1.058	SLC3A1, PREPL, CAMKMT, ENSBTAG00000040564, ENSBTAG00000043226	2
12	78,134,904	78,134,904	3.16 × 10^−7^	0.536	ITGBL1, 5S_rRNA, FGF14	1
12	78,813,208	78,813,208	4.52 × 10^−7^	0.338		1
13	16,268,793	16,268,793	5.32 × 10^−8^	1.223	ITIH5	1
15	65,654,411	65,654,411	9.20 × 10^−8^	1.138	CD44	1
16	44,888,428	45,019,492	7.68 × 10^−10^	0.613	RERE, bta-mir-2285ck, SLC45A1	2
16	46,077,301	47,047,708	2.77 × 10^−8^	0.619	DNAJC11, THAP3, PHF13, KLHL21, ZBTB48, TAS1R1, NOL9, PLEKHG5, TNFRSF25, ESPN, HES2, ACOT7, GPR153, ENSBTAG00000054938, ENSBTAG00000049238	7
16	72,020,440	72,020,440	4.42 × 10^−7^	1.127	RD3, TRAF5, RCOR3	1
16	74,098,459	74,098,459	4.52 × 10^−7^	0.598		1
17	8,929,084	8,929,084	2.27 × 10^−9^	1.338		1
17	57,930,217	58,393,881	3.13 × 10^−8^	0.338	FBXO21, TESC, FBXW8, RNFT2, SPRING1, U6, ENSBTAG00000053074, ENSBTAG00000037415, ENSBTAG00000051326, ENSBTAG00000053055	3
20	37,487,725	37,487,725	2.18 × 10^−7^	1.105		1
20	38,295,510	38,295,510	5.32 × 10^−8^	1.223	CAPSL, IL7R	1
21	13,584,472	13,584,472	2.27 × 10^−9^	1.338		1
21	58,429,839	58,429,839	1.03 × 10^−7^	0.922	PRIMA1	1
22	12,939,505	12,984,148	3.85 × 10^−7^	0.502	MYRIP, ENSBTAG00000049890	2
22	28,603,524	28,603,524	2.00 × 10^−7^	0.615		1
22	33,464,942	33,464,942	5.60 × 10^−8^	0.553	TAFA1	1
25	12,368,203	12,394,807	5.32 × 10^−8^	1.223		3
26	8,076,021	8,076,021	1.36 × 10^−7^	0.511	PRKG1	1
26	12,482,172	12,499,709	2.18 × 10^−7^	1.105	HTR7, RPP30, ANKRD1	2
26	15,176,321	15,176,321	8.37 × 10^−11^	0.994	5S_rRNA, ASMTL, SLC25A6, ENSBTAG00000051075, ENSBTAG00000052863, ENSBTAG00000052720, ENSBTAG00000055018	1
28	38,626,680	38,626,680	2.57 × 10^−7^	0.675		1

^1^ Chromosome QTL location. ^2^ Candidate genes within the identified QTL.

**Table 2:** Results of the pathway enrichment analysis using the candidate genes associated with high IFNɣ production.

ID	Description	Adjusted *p*	Gene Code	Gene Ratio
bta04217	Necroptosis	0.008946	PLA2G4E/PLA2G4D/PLA2G4F/ TRAF5/SLC25A6/MAPK9	6/42
bta04730	Long-term depression	0.008946	PLA2G4E/PLA2G4D/PLA2G4F/ PRKG1	4/42
bta04611	Platelet activation	0.008946	PLA2G4E/PLA2G4D/PLA2G4F/ SNAP23/PRKG1	5/42
bta04664	Fc epsilon RI signaling pathway	0.008946	PLA2G4E/PLA2G4D/PLA2G4F/ MAPK9	4/42
bta00592	alpha-Linolenic acid metabolism	0.008946	PLA2G4E/PLA2G4D/PLA2G4F	3/42
bta04912	GnRH signaling pathway	0.017492	PLA2G4E/PLA2G4D/PLA2G4F/ MAPK9	4/42
bta04014	Ras signaling pathway	0.017492	PLA2G4E/PLA2G4D/PLA2G4F/ HTR7/FLT4/MAPK9	6/42
bta00591	Linoleic acid metabolism	0.017492	PLA2G4E/PLA2G4D/PLA2G4F	3/42
bta05231	Choline metabolism in cancer	0.017492	PLA2G4E/PLA2G4D/PLA2G4F/ MAPK9	4/42
bta04750	Inflammatory mediator regulation of TRP channels	0.023268	PLA2G4E/PLA2G4D/PLA2G4F/ MAPK9	4/42
bta00565	Ether lipid metabolism	0.025462	PLA2G4E/PLA2G4D/PLA2G4F	3/42
bta04726	Serotonergic synapse	0.026458	PLA2G4E/PLA2G4D/PLA2G4F/ HTR7	4/42
bta04370	VEGF signaling pathway	0.026458	PLA2G4E/PLA2G4D/PLA2G4F	3/42
bta04913	Ovarian steroidogenesis	0.031114	PLA2G4E/PLA2G4D/PLA2G4F	3/42
bta04270	Vascular smooth muscle contraction	0.037248	PLA2G4E/PLA2G4D/PLA2G4F/ PRKG1	4/42

## Data Availability

The original contributions presented in this study are included in the article/Supplementary Materials. Further inquiries can be directed to the corresponding author. Sequence data used in this study for the imputation to WGS are owned by the 1000 Bull Genomes Project Consortium. Individual genotype data used in this study are managed by a third party, the Spanish Friesian Cattle National Federation (CONAFE). Request for individual genotype data can be made to CONAFE, Ctra. De Andalucia, km. 23,600-28340 Valdemoro, Madrid, Spain; email: conafe@conafe.com; phone: +34-(91)-8952412; Fax: 918-951-471; website: www.conafe.com. CONAFE is a member of the Eurogenomics Cooperative U.A.

## Supplementary Materials

The following supporting information can be downloaded at https://www.mdpi.com/article/10.3390/microorganisms11071817/s1: Table S1. IFNɣ levels (OD values) after blood stimulation with aPPD, bPPD, and PBS; Table S2. Overlapping of the identified QTLs with QTLs associated with other health and reproductive traits; Table S3. Enriched QTLs identified in the QTL enrichment analysis.
